# Seroepidemiology of Chagas disease in at-risk individuals in Caraíbas, a city with high endemicity in Bahia State, Brazil

**DOI:** 10.3389/fpubh.2023.1196403

**Published:** 2023-09-22

**Authors:** Tycha Bianca Sabaini Pavan, Deorlan Pereira Dias, Márcia Moraes Cangussú, Vilquenia Porto Pacheco Dutra, Daniel Dias Sampaio, Fred Luciano Neves Santos

**Affiliations:** ^1^Advanced Public Health Laboratory, Gonçalo Moniz Institute, Oswaldo Cruz Foundation (Fiocruz-BA), Salvador, Brazil; ^2^Chagas Disease Working Group, Municipal Health Department, Center for Endemic Diseases, Caraíbas, Brazil; ^3^Bahia State Department of Health, Southwest Regional Health Center, Vitória da Conquista, Brazil; ^4^Brazil’s Family Health Strategy, Municipal Health Department, Tremedal, Brazil; ^5^Integrated Translational Program in Chagas Disease from Fiocruz (Fio-Chagas), Oswaldo Cruz Foundation (Fiocruz-RJ), Rio de Janeiro, Brazil

**Keywords:** chronic Chagas disease, screening, serologic diagnosis, prevalence, Caraíbas, seroepidemiology, active case finding, epidemiological surveillance

## Abstract

**Introduction:**

In Brazil, an estimated 1.1 million people are infected with *Trypanosoma cruzi*, the causative agent of Chagas disease (CD). Despite the high number of cases, the estimated prevalence of infection per 100 inhabitants is low (0.03). However, the actual number of chronically infected individuals is still unknown. Therefore, we sought to determine the prevalence of chronic CD in at-risk individuals in Caraíbas (Bahia, Brazil) through active case finding.

**Methods:**

A total of 572 individuals living in rural or urban areas of Caraíbas were eligible for the study. A serum sample was collected from 226 individuals, and the diagnosis performed according to international guidelines.

**Results:**

The overall prevalence of anti-*T. cruzi* IgG was 4.42%. The median age of anti-*T. cruzi* IgG-positive individuals was 54.5 years, and the female-to-male ratio was 1.5:1. The prevalence of anti-*T. cruzi* IgG was similar in rural (4.29%) and urban areas (4.65%).

**Discussion:**

Compared with national estimates, we concluded that Caraíbas had a high prevalence for chronic CD and a high risk for persistent transmission. Through our study, it was possible to monitor individuals who were unaware of their clinical condition, thus improving their quality of life.

## 1. Introduction

Chagas disease (CD) is a neglected vector-borne life-threatening infection caused by the protozoan hemoflagellate *Trypanosoma cruzi*. In endemic areas, the parasite is usually transmitted to humans through contact with contaminated feces or urine from blood-sucking triatomine insects, also known as “kissing bugs,” which are widely distributed from the southern United States to Argentina (1). In addition to transmission by vectors, the parasite can also be transmitted by other routes. These include transmission from oral ingestion of contaminated beverages or food, mother to child, transfusion of contaminated blood or blood products, bone marrow and organ-derived transmission, and, less commonly, laboratory accidents (2). The disease represents the largest burden of parasitic disease in 21 Latin American countries, where an estimated 5.7 million people are affected (1), resulting in an annual loss of more than 800,000 disability-adjusted life years (3). It is estimated that 62.4% of affected people live in the Southern Cone, but Argentina, Brazil, and Mexico are the three countries with the highest estimated number of infected people (1,505,235, 1,156,821, and 876,458, respectively), followed by Bolivia (607,186) (1). Data from WHO show that approximately 7,500 CD-associated deaths are reported annually. Due to the constant presence of the vector, 70.2 million people in endemic countries are at risk of infection (1). Increasing international migration flows and more favorable travel conditions have contributed to the spread of CD to non-endemic areas, including North American, European, Asian, and Oceanic countries (4–7).

In Brazil, the country where *T. cruzi* was first described (8), CD is a massive public health problem. WHO estimates that 1.1 million people are infected with *T. cruzi* and 25.4 million people are at risk of infection (1). Despite the high number of cases in Brazil, the estimated prevalence of *T. cruzi* infection per 100 inhabitants is low (0.03) compared to other south American endemic countries: Ecuador (1.38 cases per 100 inhabitants), Paraguay (2.13 cases per 100 inhabitants), and Bolivia (6.10 cases per 100 inhabitants) (1). The first national serological survey, conducted in Brazil between 1975 and 1980, showed that 36% of the national territory was at risk for vector transmission, with triatomine insects present in 2,493 cities in 18 Brazilian states. The prevalence of infection in the country’s rural population was estimated at 4.2%, with higher rates in Bahia (5.4%), Goiás (7.4%), Minas Gerais (8.8%), Rio Grande do Sul (8.8%) (9). A second (and last) national serological survey was conducted in 2001–2008 in a representative sample of individuals aged 5 years and younger (104,954 children) in all rural areas of Brazil except the state of Rio de Janeiro (10). Infection was confirmed in only 32 cases, representing a low nationwide prevalence (0.03%). The results of the last national serological survey show that the regular and systematic control programs against CD transmission, together with the socioeconomic advances observed in Brazil in recent decades, have interrupted vector-borne transmission in Brazil, which has resumed in the few cases.

Despite progress in interrupting CD transmission in Brazil, the actual number of chronically infected individuals is still unknown. Health policies are programmed based on estimates, which does not reflect reality given the size and diversity of the country. Therefore, studies based on active case finding (ACF) should be continuously promoted as a strategy to detect chronic CD cases in the Brazilian population, even if they are conducted in small areas. Considering the predicaments herein set forth, we sought to determine the prevalence of chronic CD among people at risk of infection in Caraíbas, an endemic city in the Brazilian state of Bahia.

## 2. Materials and methods

### 2.1. Study area

**Figure 1:**
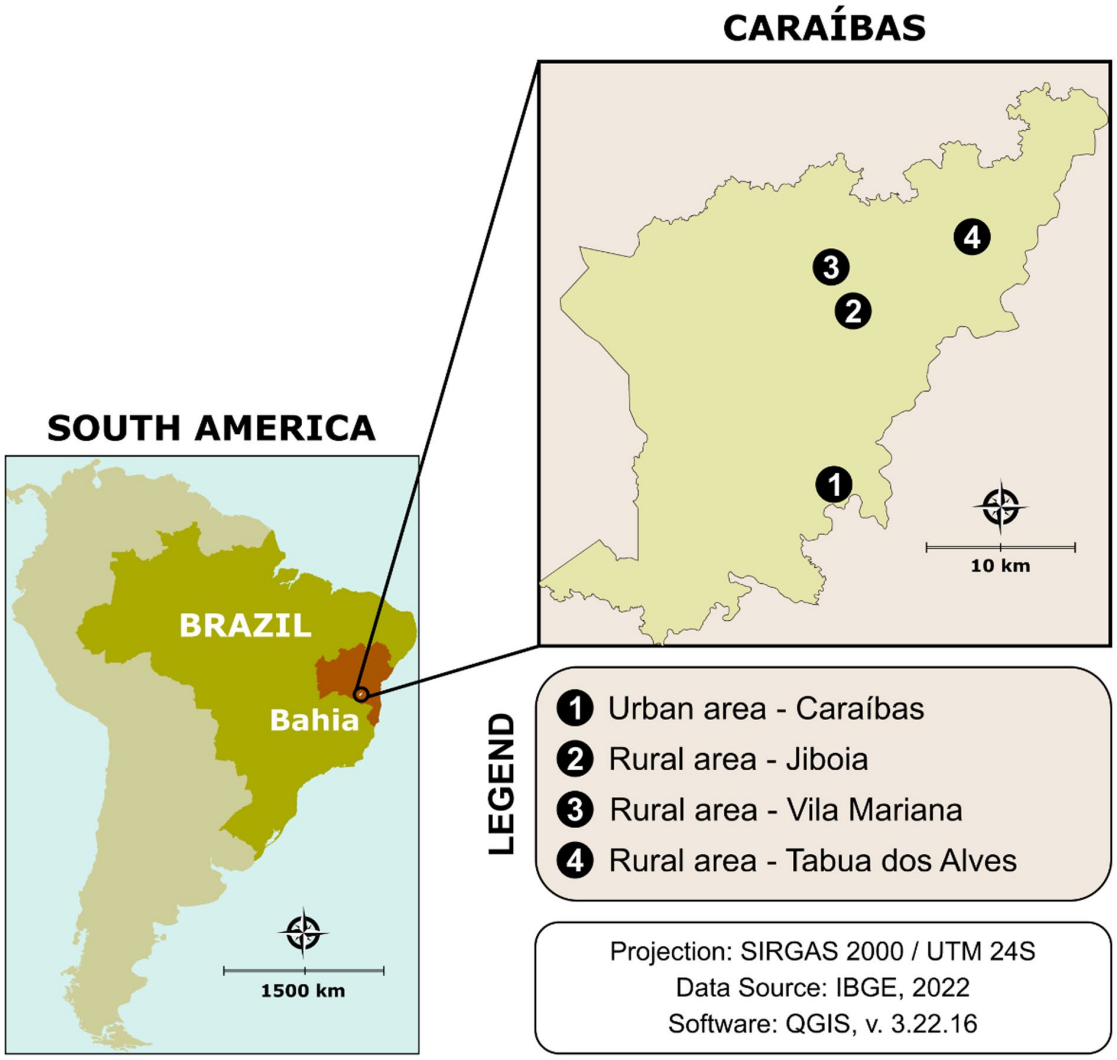
Geographic location of sample collection sites in this study. Public domain digital map was freely obtained from the Brazilian Institute of Geography and Statistics (IBGE) cartographic database in shapefile format (.shp), which was subsequently reformatted and analyzed using QGIS version 3.22.16 (Geographic Information System, Open-Source Geospatial Foundation Project. http://qgis.osgeo.org).

The present study was conducted in Caraíbas (S 14°36′41″/W 41°20′06″; Figure 1), a CD endemic municipality in the southwestern of the state of Bahia, approximately 585 km from Salvador, the capital of Bahia. The municipality has an area of 805,629 km^2^ and an estimated population of 9,940 inhabitants, with a population density of 12.34 inhabitants/km^2^. *Per capita* income is low, ranking 5,556th among the 5,570 Brazilian municipalities and 417st among the 417 municipalities in Bahia (11). The population is distributed between urban (~15%) and rural villages (Vila Mariana, Jiboia and Tabua dos Alves). Rural areas are generally characterized by poor housing quality, with the presence of domestic animal breeding sites, such as perches, pigsties, corrals, and barns, which favor the development of triatomines. Caraíbas has a semi-arid climate and characteristic semi-arid tropical vegetation (caatinga) (12), which favors the formation of ecotopes for the development of local triatomine species, such as *Panstrongylus* spp. and *Triatoma* spp.

### 2.2. Study design

We conducted a cross-sectional epidemiological study in four distinct areas: three rural zones (Vila Mariana, Jiboia, and Tabua dos Alves) and the urban region of Caraíbas. The selection of these sites was guided by specific criteria, including the presence of *T. cruzi*-infected individuals in the population, the existence of mud houses with cracks, residences with proximity to domestic animals (chickens), houses situated near forested areas, dwellings where triatomines had been previously captured (regardless of infection status), and the presence of individuals with constipation or heart disease. No additional inclusion or exclusion criteria were applied. Utilizing these parameters, community health officials identified and enrolled 572 individuals residing in high-risk areas for vector transmission who met the study’s eligibility requirements.

### 2.3. Study population

The study encompassed individuals of both genders, aged 6 years or older, who were registered at public primary health care (PHC) facilities in both the rural and urban areas of Caraíbas. Our research team conducted in-person visits to eligible individuals and invited them to partake in the study, subject to obtaining written informed consent. Subsequent to enrollment, participants were interviewed regarding potential signs and symptoms of CD, and blood samples were collected for serological diagnosis. In cases where initial contact with eligible individuals could not be established, up to five further attempts were undertaken. In addition to assessing the serological status for CD, other variables, such as self-reported skin color, age group, and years of education, were analyzed. Self-reported skin color was classified into the following categories: European ancestry, dark skin, East Asian ancestry, indigenous ancestry, and mixed-race (individuals whose skin color did not fit into the categories of dark-skinned, European, indigenous, or East Asian).

### 2.4. Serology testing

Blood samples were collected by nurse assistants. Five ml of peripheral blood was drawn from each participant. Samples were allowed to stand at room temperature for 30 min to allow clotting and then centrifuged at 3,000 g for 15 min. The serum was separated and frozen at −20°C until further use. The serum samples were sent to the Central Public Health Laboratory of Bahia (LACEN-BA) and total anti-*T. cruzi* antibodies were detected by two immunoassays with different methodological principles (13, 14): an enzyme immunoassay (Biolisa Chagas Recombinante, Bioclin, Belo Horizonte-MG, Brazil; or Anti-Chagas SYM, Vyttra Diagnósticos, São Paulo-SP, Brazil) and a chemiluminescence assay (Architect Chagas, Abbott Laboratories, Chicago-IL, United States), according to the manufacturer’s instructions. In case of disagreement, a third method was used (indirect immunofluorescence; IFI-Chagas-Bio-Manguinhos, Fiocruz, Rio de Janeiro-RJ, Brazil). Participants received their serology results through a physician at a PHC facility.

### 2.5. Sample size and statistical analysis

The sample size was determined using the Wald method (15), assuming an infinite population with a 95% confidence interval (95% CI), an absolute error of 6%, and an estimated prevalence of CD in the state of Bahia of 20.4% (16). Based on these parameters, obtained using OpenEpi, a free web-based open-source program (17), the minimum sample to conduct this study was 174 individuals. A total of 226 (39.5%) individuals living in rural (Vila Mariana, Jiboia, and Tabua dos Alves) and urban areas of Caraíbas were randomly chosen by drawing lots from 572 individuals previously selected for the study by community health officials, and sera were obtained for serological testing. Patient characteristics and results were recorded in an encrypted electronic data collection database. Categorical variables were described with frequencies and percentages and continuous variables with means, standard deviations, and ranges. We utilized the chi-square test to compare the prevalence between urban and rural settings. Analysis was performed with MedCalc software.^1^

## 3. Results

A total of 226 individuals participated in this study. Their attributes are shown in Table 1. The median age was 53 years (interquartile range [IQR] 38–64), with more women (132; 58.4%) than men (94; 41.6%) participating. Compared with the general Brazilian population (18), a smaller proportion of participants were between their 10s and 30s (26.1% vs. 58.5% in the study and general population, respectively) and ≥70 years (14.2% vs. 7.1%), whereas more participants were between their 40s and 60s (59.7% vs. 34.4%). The proportion of individuals who lived in rural areas (140; 61.9%) was higher than those who lived in urban areas (86; 38.1%).

**Table 1:** Demographic characteristics of the study participants living in urban and rural areas of the municipality of Caraíbas-BA.

	Vila Mariana^a^ (*n* = 55) *n* (%)	Jiboia^a^ (*n* = 51) *n* (%)	Tabua dos Alves^a^ (*n* = 34) *n* (%)	Caraíbas^b^ (*n* = 86) *n* (%)	Total (*N* = 226) *N* (%)
**Gender**					
Female	28 (50.9)	29 (56.9)	21 (61.7)	54 (62.8)	132 (58.4)
Male	27 (49.1)	22 (43.1)	13 (38.3)	32 (37.2)	94 (41.6)
**Age in years**					
0–9	0	0	0	1 (1.2)	1 (0.4)
10–19	0	6 (11.8)	1 (2.9)	5 (5.8)	12 (5.3)
20–29	2 (3.6)	2 (3.9)	7 (20.6)	9 (10.5)	20 (8.8)
30–39	10 (18.2)	6 (11.8)	2 (5.9)	8 (9.3)	26 (11.5)
40–49	8 (14.6)	7 (13.7)	7 (20.6)	17 (19.7)	39 (17.3)
50–59	10 (18.2)	13 (25.5)	3 (8.8)	25 (29.0)	51 (22.7)
60–69	18 (32.7)	9 (17.6)	8 (23.6)	10 (11.6)	45 (19.9)
70–79	6 (10.9)	7 (13.7)	5 (14.7)	9 (10.5)	27 (11.9)
80–89	1 (1.8)	1 (2.0)	1 (2.9)	1 (1.2)	4 (1.8)
90–99	0	0	0	1 (1.2)	1 (0.4)

a Rural areas.

b Urban area.

**Figure 2:**
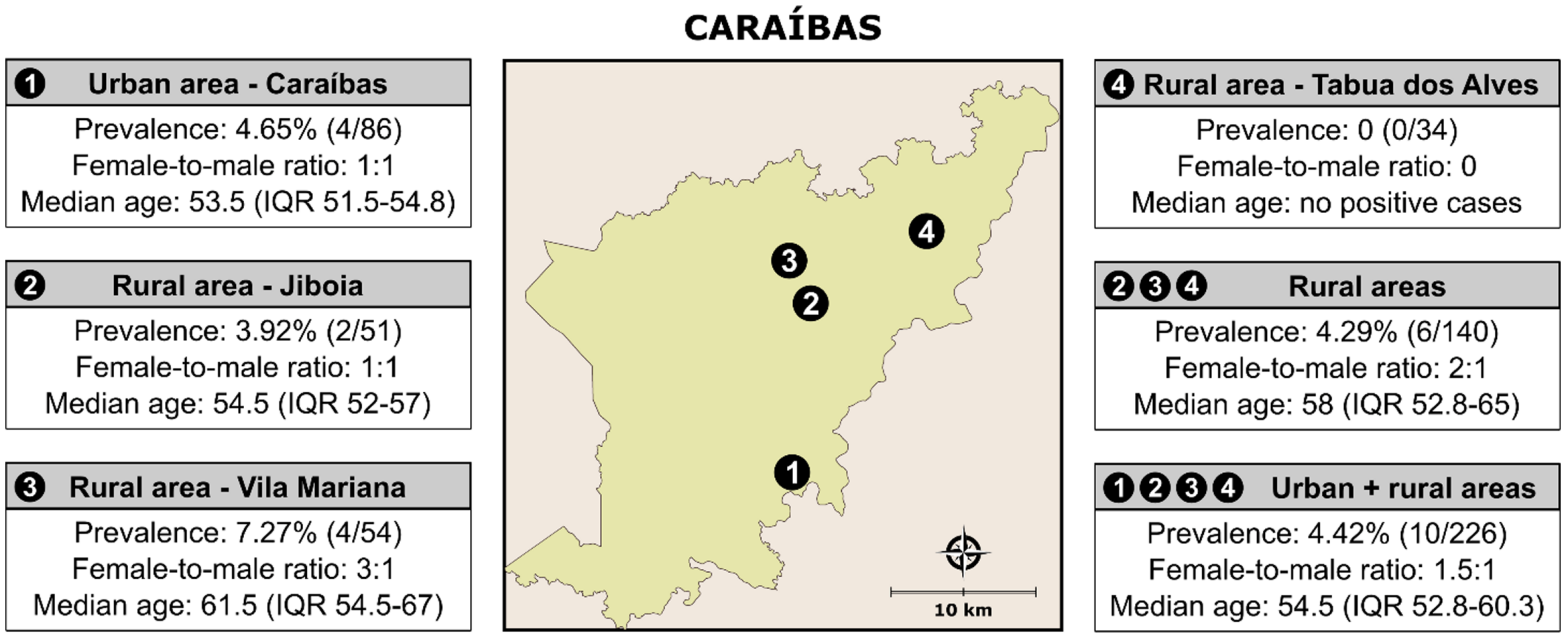
Prevalence of anti-*T. cruzi* IgG in individuals living in rural and urban areas of the municipality of Caraíbas, Bahia. Public domain digital map was freely obtained from the Brazilian Institute of Geography and Statistics (IBGE) cartographic database in shapefile format (.shp), which was subsequently reformatted and analyzed using QGIS version 3.22.16 (Geographic Information System, Open-Source Geospatial Foundation Project. http://qgis.osgeo.org).

The overall prevalence of anti-*T. cruzi* IgG was 4.42% (10/226). Figure 2 provides the prevalence data for anti-*T. cruzi* IgG in the different demographic situations. The median age of anti-*T. cruzi* IgG-positive individuals was 54.5 years (IQR 52.8–60.3) and the female-to-male ratio was 1.5:1. The median age was 55.5 years (IQR 53–60.3) for women and 53.5 years (IQR 51.33–64.8) for mem (*p* = 0.593). Self-reported skin color showed that 80% identified themselves as “mixed-race” and 20% had European ancestry. Regarding education level, the majority of participants (80%) had up to 8 years of formal schooling and 20% were illiterate. Out of the 10 *T. cruzi*-positive-participants with available income information, approximately 20% reported an annual personal income until USD 1450 and, the other 80% reported a mean of USD 3200. The prevalence of anti-*T. cruzi* IgG was similar in rural (4.29%; 6/140) and urban areas (4.65%; 4/86) (*p* = 0.029). In rural areas, we observed a higher prevalence in Vila Mariana (7.27%; 4/55), followed by Jiboia (3.92%; 2/51). No cases were observed in Tabua dos Alves.

Table 2 shows the characteristics of the 10 anti-*T. cruzi* IgG-positive individuals. In two cases, it was reported that a sibling in the family had also been diagnosed with CD. An electrocardiogram was performed in half of the patients, and changes were noted in three of them: Right and left bundle branch block in a 53-year-old woman from Vila Mariana; left ventricular overload and nonspecific change in ventricular repolarization in a 57-year-old woman from Jiboia; and sinus bradycardia associated with a prolonged QT wave in a 52-year-old man also from Jiboia. Regarding digestive symptoms, only two cases were diagnosed radiologically without changes.

**Table 2:** Demographic characteristics of anti-*T. cruzi* IgG-positive individuals living in urban and rural areas of the municipality of Caraíbas-BA.

Cases	Sampling location	Sex	Age	CD-positive family member	Electrocardiography (ECG)	Colonoscopy
#1	VM	F	53	Sibling	Right bundle branch block + left bundle branch block	NP
#2	VM	F	59	No	NC	NP
#3	VM	F	64	No	NP	NP
#4	VM	M	68	No	NP	NP
#5	CA	F	54	Sibling	NC	NC
#6	CA	F	53	No	NP	NC
#7	CA	M	55	No	NP	NP
#8	CA	M	51	No	NP	NP
#9	JI	F	57	No	Left ventricular overload + nonspecific changes in ventricular repolarization	NP
#10*	JI	M	52	No	Sinus bradycardia + prolonged QT wave	NP

*Individuals using a pacemaker. CA, Caraíbas; F, Female; JI, Jiboia; M, Male; NC, No changes; NP, Not performed; VM, Vila Mariana.

## 4. Discussion

In this study, the seroepidemiological situation of Chagas disease in rural and urban areas of Caraíbas was investigated, with serodiagnosis performed in approximately 2.2% of the inhabitants of these areas. In Brazil, significant progress has been achieved in recent decades in the fight against CD. In fact, the number of vector-borne cases has decreased to a few cases in recent years (2, 10). However, despite the decrease in new cases, the improvement in the quality of life of the population, the introduction of hemovigilance in blood centers, and the progress in prevention campaigns, the number of chronic cases remains unknown. Data from WHO show that 20.1% of the 5,742,167 chronic CD cases estimated for the 21 Latin American cases live in Brazil (1). In terms of the absolute number of CD chronic cases, Brazil ranks second in the world, surpassing only Argentina. Efforts to obtain these estimates are valuable, but they do not reflect local realities. Brazil is a continental-sized country with a diversity of climates, ecotopes, vegetation, and cultures. Therefore, studies based on active case finding (ACF) should be promoted as a strategy to detect chronic CD cases in the Brazilian population, even if they are conducted in small areas such as Caraíbas, an endemic city in the Brazilian state of Bahia. In the present study, we found a prevalence of anti-*T. cruzi* IgG in 4.42% of the study population of Caraíbas.

In a systematic review and meta-analysis study published in 2014, the mean prevalence for Brazil was reported to be 4.2%, considering published data from 1980 to 2011 from 18 Brazilian states. In the same study, a mean prevalence of 20.4% was calculated for the state of Bahia based on two available studies. The first study was conducted in Catolândia in 1989, in which infection was detected in 11.1% of 344 individuals aged 0 to >61 years (19). The second study was conducted in 2002 in Mulungu do Morro, where 25.1% of 694 individuals tested positive for CD (20). In the present study, we found a prevalence of 4.42%, a value that corresponds to the mean prevalence estimated for Brazil and is lower than in Catolândia and Mulungu do Morro. The discrepancy between Mulungu do Morro/Catolândia and our study is probably due to the improvement in the quality of life of the population and the progress in prevention campaigns against triatomines. Indeed, the social programs implemented by the Brazilian government in the last 20 years have had a positive impact on the quality of life of the population (21, 22). Despite this significant decrease, the high prevalence (4.42%) of CD in this study can be explained by the higher sensitivity and specificity of the recently used methods (23, 24). On the other hand, our results are consistent with the seroprevalence in the Brazilian states of Ceará (Quixeré, 3.7% 13/348 and Limoeiro do Norte, 4.2% 34/812) (25, 26), Piauí (Campinas do Piauí; 5.8% 44/763) (27), and Amazonas (Rio Negro; 5.2% 25/482) (28).

In our study, all positive individuals were older than 50 years, showing the aging of the CD population, which has also been found in other studies (19, 20, 25–27). Approximately 14.6% of our samples were from individuals aged 30 years or younger, and all were negative for anti-*T. cruzi* IgG. This result could be an indication of the effectiveness of vector control and improvement of housing conditions in rural and urban areas of Caraíbas. However, entomological surveys conducted by local health authorities indicate that conditions remain favorable for the maintenance of Chagas endemicity in Caraíbas. In the distribution by sex, we observed a similar positivity in males and females. Also, no difference was observed in the prevalence of anti-*T. cruzi* IgG in individuals living in rural (4.29%) or urban areas (4.65%). In this study, individuals who tested positive for *T. cruzi* were predominantly found within the economically disadvantaged mixed-race population (earning less than US$ 3200.00 per year), highlighting the connection between Chagas disease and poverty. Our results are consistent with those described for individuals from Mulungu do Morro, Bahia (20).

In two diagnosed cases, vertical transmission was probably the cause, as other positive family members were mentioned. However, because we did not have access to the mother to confirm the transmission was congenital. Therefore, vectorial transmission must be considered the main route of transmission, because the positive cases did not report having a mother with CD or having donated or received blood before the study. Electrocardiography or colonoscopy was performed in some positive cases. Only two individuals who underwent colonoscopy were found to have no digestive disturbances. Electrocardiography was performed in five cases, and changes were found in three patients. One of them wears a pacemaker. These findings suggest that the infection in these individuals occurred a long time ago, suggesting vectorial transmission in childhood or less relevant congenital transmission.

The main limitation of the present study is the lack of representativeness of the community. Indeed, the sampling was based on criteria that prioritized the population at risk for *T. cruzi* infection. The total population of the community was not considered in determining the study population due to the limited number of tests planned for the study. Another limitation is the lack of electrocardiographic and colonoscopic testing in *T. cruzi*-infected individuals. Despite these limitations, our study identified new cases of CD and provided medical care and follow-up to some individuals who were not yet aware of their clinical status.

We concluded that Caraíbas has a high prevalence for chronic CD and a high risk for persistent transmission. Through our study, it was possible to monitor individuals who were unaware of their clinical condition, thus improving their quality of life. In addition, our study, although not population-based, showed that local prevalence is lower than estimates for the state of Bahia, which could support measures for better use of public resources in programs to control transmission.

## Data availability statement

The original contributions presented in the study are included in the article/supplementary material, further inquiries can be directed to the corresponding author.

## Ethics statement

The studies involving humans were approved by Institutional Review Board (IRB) for Human Research at the Gonçalo Moniz Institute, Oswaldo Cruz Foundation (FIOCRUZ). The studies were conducted in accordance with the local legislation and institutional requirements. The participants provided their written informed consent to participate in this study.

## Author contributions

TP, DD, MC, VD, DS, and FS contributed substantially to the work described in this article. MC, VD, and FS designed the experimental procedure. DD and VD supervised the field activities. TP, DS, and FS performed the statistical analysis. TP and FS wrote the article. DD, MC, VD, and DS helped write the article. TP, VD, DS, and FS performed data collection, analysis, and interpretation. MC provided the serological diagnosis. FS produced the illustrations and obtained funding for this study. FS supervised the work. All authors contributed to the article and approved the submitted version.

## Footnotes

^1^https://www.medcalc.org
